# Adenocarcinoma with adenoma in the jejunum suggesting an adenoma-carcinoma sequence in the small bowel: A case report

**DOI:** 10.3892/ol.2014.2210

**Published:** 2014-06-02

**Authors:** YORIKA NAKANO, YASUSHI ADACHI, HIDEKI OKAMOTO, YOSHIAKI KIYAMA, TAKASHI KOYAMA, SHIN-ICHI NAKAMURA, QING LI, NORIKO SAKAIDA, YOSHIKO UEMURA, SUSUMU IKEHARA

**Affiliations:** 1 Department of Surgical Pathology, Kansai Medical University-Hirakata Hospital, Osaka 573-1191, Japan; 2 Division of Surgical Pathology, Toyooka Hospital, Hyogo 668-8501, Japan; 3 Department of Stem Cell Disorders, Kansai Medical University, Osaka 573-1010, Japan; 4 Department of Surgery, Asago-Yanase Medical Center, Hyogo 669-5103, Japan; 5 Department of Radiology, Toyooka Hospital, Hyogo 668-8501, Japan; 6 LSI Medience Corporation, Tokyo 174-0051, Japan

**Keywords:** adenocarcinoma, adenoma, jejunum, small bowel, p53, Ki-67

## Abstract

Other than that in the duodenum, adenocarcinoma in the small bowel is rare. The present study describes a case of adenocarcinoma with adenoma in the jejunum. A 70-year-old male was admitted to hospital due to dehydration induced by abdominal discomfort and difficulty with oral intake. Computed tomography revealed a tumor in the upper side of the jejunum, which was subsequently resected. The tumor contained adenocarcinoma and adenoma. The protein expression of p53 and Ki-67 was analyzed in the normal mucosa, adenoma and adenocarcinoma. The number of epithelial cells expressing p53 and Ki-67 was found to increase in the adenoma tissue compared with that in the normal mucosa. In the adenocarcinoma tissue, the number of cells expressing p53 and Ki-67 further increased, suggesting that an adenoma-adenocarcinoma sequence may occur in the small bowel, similar to that observed in the large bowel.

## Introduction

Primary small bowel adenocarcinoma (PSBA) is rare and arises in the duodenum in more than a half of cases (1). Thus, adenocarcinoma in the jejunum is extremely rare. It has been reported that PSBA accounts for ~2% of gastrointestinal tumors and that PSBA originates in the duodenum in 272/523 (52.0%), jejunum in 98/523 (18.7%), ileum in 90/523 (17.2%), and unspecified sites in 63/523 (12.0%) of patients (1). Clinical symptoms of PSBA include abdominal pain, bleeding, vomiting, loss of appetite and jaundice (2). For the treatment of PSBA, resection of the tumor, chemotherapy and radiation are performed alone or in combination (2). If it is in the early stage, endoscopic mucosal resection is also performed. The prognosis for PSBA is poor, predominantly due to the delay in detection. The present study describes a case of advanced adenocarcinoma with adenoma in the jejunum of an elderly male. The family of the patient provided written informed consent. This study was approved by the Ethics Committee of Toyooka Hospital (Toyooka, Japan).

## Case report

### Case presentation

**Figure 1:**
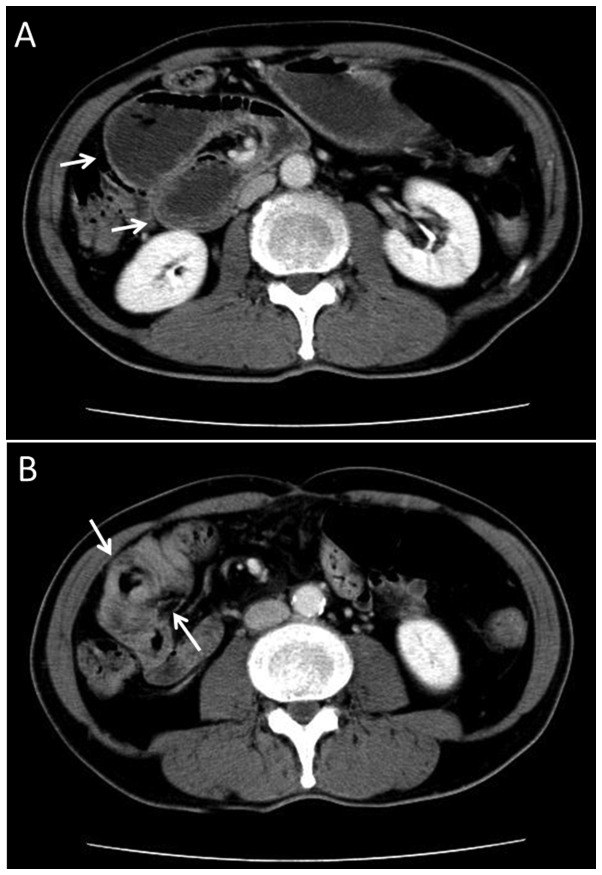
Abdominal contrast CT scan of the patient showing (A) dilatation of the duodenum and the upper side of the jejunum (arrows) and (B) a tumor in the upper side of the jejunum. The tumor was present in almost the entire circumference of the jejunal wall (arrows). CT, computed tomography.

A 70-year-old male presented at the Asago-Yanase Medical Center, (Asago, Japan) due to upper abdominal discomfort and dehydration lasting for three months. Contrast computed tomography scans revealed severe dilatation of the stomach, duodenum and upper section of the jejunum, and also revealed a tumor in the jejunum (Fig. 1). The tumor had induced wall thickening around almost the entire circumference of the jejunum at about 50 mm towards the anus from the ligament of treitz. Serologically, the carcinoembryonic antigen and carbohydrate antigen 19-9 titers were found to be within the normal limits.

### Tumor resection

**Figure 2:**
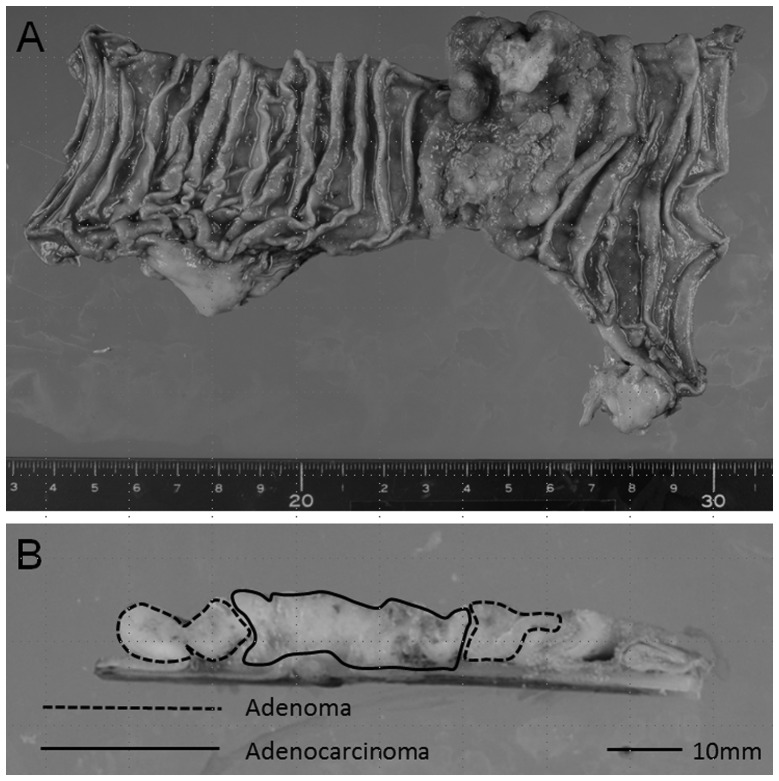
Macroscopic observation of the resected tumor. (A) The formalin-fixed resected tumor. (B) The adenoma (dotted line) and adenocarcinoma (solid line) areas in the vertical section of the tumor.

**Figure 3:**
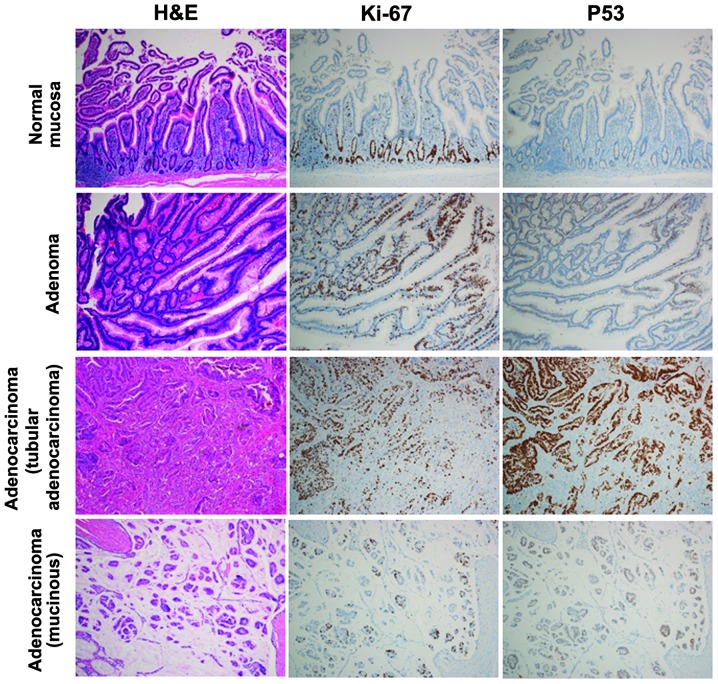
Microscopic analysis of the tumor showing representative H&E-stained sections, as well as p53 and Ki-67 expression in the normal mucosa, adenoma, well- and moderately differentiated adenocarcinoma, and mucinous adenocarcinoma (magnification, ×10). H&E, hematoxylin and eosin.

The tumor was resected and a pathological examination of the tumor was performed. Macroscopically, the tumor was 60×52 mm and was classified as Borrmann type 2 (Fig. 2A). Histologically, the tumor contained areas of adenoma and adenocarcinoma tissue (Figs. 2B and 3). The adenocarcinoma was found to be in the center of the tumor, while the adenoma was at the edge (Fig. 2B). In the adenocarcinoma area, the carcinoma showed various differentiating subtypes of adenocarcinoma, including mucinous adenocarcinoma, well-differentiated tubular adenocarcinoma, moderately differentiated tubular adenocarcinoma and poorly differentiated adenocarcinoma. Furthermore, the tumor had invaded the subserosa and had metastasized into the regional lymph nodes.

### Immunohistological analysis

The tumor was immunohistologically analyzed for the expression of P53 and Ki-67. As shown in Fig. 3, in the normal mucosa of the jejunum, there were no P53-expressing epithelial cells and the Ki-67-expressing epithelial cells primarily formed a regular pattern at the base of the crypts. In the adenoma tissue, there was a greater number of Ki-67-expressing epithelial cells compared with the normal mucosa, and the cells were distributed in an irregular pattern. Certain adenoma cells expressed a low level of P53. In the adenocarcinoma tissue, there was a greater number of Ki-67-expressing epithelial cells compared with the adenoma tissue, and the adenocarcinoma tissue strongly expressed P53. Furthermore, in the adenocarcinoma, there were a greater number of Ki-67- and P53-expressing cells in the tubular adenocarcinoma area than in the mucinous adenocarcinoma area.

## Discussion

PSBA is a very rare condition. PSBA accounts for ~2% of all cases of gastrointestinal cancer, while colorectal tumors account for >52% of gastrointestinal carcinoma cases (3,4). Verma and Stroehlein (1) analyzed 523 cases of SBA at the MD Anderson Cancer Center over a period of 60 years between 1944 and 2003 (1). The mean age at the time of admission was 54 ± 13 years old (range, 21–87) and the male to female ratio was 58:42. With regard to the location of the tumors, 272 were duodenal, 98 were jejunal, 90 were ileal and 63 were not specified (1). Hong *et al* (2) investigated PSBA in 53 patients in Korea. The mean age of the patients was 63 years (range, 26–84), with 79.3% being >50 years old. The male to female ratio was 54.7:43.3 and 73.6% of the tumors were located in the duodenum, 13.2% in the jejunum and 13.2% in the ileum (2). Misawa *et al* (5) also analyzed 116 cases of PSBA in the jejunum and ileum between 2005 and 2010 in Japan (5). The mean age of the patients was 60.8 years old, and the male to female ratio was 65:35. With regard to the location of the tumor, 54% were located in the jejunum, while 46% were located in the ileum. These studies all showed a similar pattern, with the duodenum being the main site of occurrence and with more adenocarcinomas arising in the duodenum than in the jejunum and ileum combined. Furthermore, the mean age of the patients at the time of admission was 50–60s and the male to female ratio was male dominant (1,2,5). In the present case, the patient was in his 70s and male, which is consistent with the previous reports.

Adenoma of the small bowel was also reported by Perzin and Bridge (6), who analyzed 392,000 surgical pathology cases over a 62-year period. A total of 51 cases of small bowel tumor containing adenomatous epithelium were found. Among the 51 patients, 18 had adenomas and 33 had tumors that contained adenoma and carcinoma (6). These findings suggest that the adenoma-adenocarcinoma sequence may occur in the small bowel as well as in the colon and rectum. In 42 cases, the tumors arose in the duodenum or consisted of multiple lesions, including duodenal lesions, while six were in the jejunum and three were in the ileum. Among the six jejunal cases, the adenocarcinoma existed with adenoma in four of the cases and without adenoma in two of the cases. In the four cases in which the carcinoma and adenoma coexisted, one case exhibited ulcerative carcinoma with a zone of adenoma at the edge, similar to that observed in the present case.

Nishiyama *et al* (7) and Arai *et al* (8) reported that 40–50% of cases of PSBA overexpress the p53 protein, suggesting that p53 may have a major role in the progression of carcinoma of the small bowel (7,8). In the present case, no p53-positive epithelial cells were found in the normal mucosa; however, p53-expressing cells were observed in the adenoma tissue and the level of p53 expression and the number of p53-expressing cells was higher in the adenocarcinoma tissue, consistent with previous findings (7,8). Moreover, in the present case, the number of Ki-67 expressing cells was observed to be higher in the adenoma tissue compared with the normal mucosa, and higher in the adenocarcinoma tissue compared with the adenoma tissue. This pattern of p53 and Ki-67 expression suggests that p53 may have a crucial role in the progression from normal mucosa to adenocarcinoma through to adenoma.

The prognosis for PSBA is poor predominantly due to delay in the detection of the tumor. However, recent developments have been made in capsule and double balloon endoscopy (9,10), and the use of a combination of these tools and imaging examinations (including computed tomography, magnetic resonance imaging and positron-emission tomography) should make it possible to detect PSBA in the early stage, allowing the tumor to be resected endoscopically.
